# Mutation Rates and Gene Location: Some Like It Hot

**DOI:** 10.1371/journal.pbio.0020051

**Published:** 2004-02-17

## Abstract

xx

The growing library of sequenced genomes is challenging scientists to extract new biological meaning from DNA sequences. Comparative analysis of the mouse and human genome, for example, has already revealed that mutation rates in the 3 billion base pairs of the human genome vary considerably. What accounts for this regional disparity, however, is unclear. Mutations—substitutions in the nucleotide bases of DNA—produce variation in the genome. In classical evolutionary theory, natural selection drives evolutionary change by determining which of these mutations live on in the next generation or die with the organism. Mutations can be neutral, harmful, or beneficial, though the neutral theory of molecular evolution predicts that most mutations are “nearly” neutral or only slightly deleterious, while beneficial mutations—which confer a survival advantage on an organism and, if it reproduces, on its progeny—are quite rare. As a whole, mutations occur at the rate of approximately five substitutions per billion nucleotide sites per year.

There are many types of neutral mutations—that is, mutations that have no effect on function. DNA base substitutions that lie outside of gene-coding regions or occur within introns (regions that are excised before being translated into a protein sequence) can fall into this category. Neutral mutations can also occur within gene-coding regions. For example, there are many instances where more than one codon—say, CUU, CUC, CUA, CUG—specify the same amino acid—in this case, leucine. Since these mutations can be used to gauge the neutral mutation rate of a region in the genome, they can be used to analyze the relationship between local mutation rates and gene location. Correlating gene mutation rates with their location in the genome, Jeffrey Chuang and Hao Li not only confirm that regional mutation rates indeed exist, but also calculate the size of these regions. Strikingly, certain classes of genes tend to congregate in mutational “hot spots”—regions with high mutation rates—while other types of genes gravitate toward “cold spots”—regions with relatively low mutation rates.

Chuang and Li first determined whether mutation rates have regional biases—that is, whether the frequency and distribution of mutations follow a distinct pattern along the genome. The researchers calculated the substitution rates of neutral mutations in nearly 15,000 orthologous mouse and human genes—orthologous genes are genes that have evolved from a common ancestor without diverging in biological function—and found that mutation rates were in fact skewed toward either high or low rates. Mutation rate analysis of the orthologs' neighbors revealed rates similarly skewed toward high or low substitutions, indicating that the region itself, rather than a particular gene, is prone to these differential rates. These regions, Chuang and Li report, were either one megabase or ten megabases long, affecting up to roughly 100 genes.

But the question remained: Does the organism take advantage of these mutational hot and cold spots? If there is an adaptive advantage, gene families should occur in an appropriate mutational zone. In mutational hot spots, for example, one would expect to find genes that would benefit from high rates of mutation, which would in turn facilitate flexible responses to constantly changing environmental stimuli. Likewise, one would expect genes in cold regions to need protection from potentially deleterious mutations. And that's just what Chuang and Li found. Overall, genes in hot regions code for proteins involved in cell signaling, such as olfactory receptors, G-protein coupled receptors, membrane proteins, and immune response proteins—being in an area subject to high mutation rates means these genes can evolve quickly enough to adapt to constantly changing stimuli. Cold-region genes code for “housekeeping” proteins involved in core cellular processes, like transcription regulation and protein modification—these genes tend to be highly conserved, changing very little since they first evolved.

Thus, it appears that natural selection may also operate at the level of gene location, relegating genes to different mutational genomic niches according to their function. While Chuang and Li explore possible mechanisms to account for these genomic niches—such as gene duplication and gene transposition—they argue that the selective pressures that influence gene location are the same that influence mutations in genes. By calculating the sizes of these mutational hot and cold regions, the researchers lay the groundwork for investigating genetic mechanisms that operate on these scales. And by showing that location matters, they have revealed a new force in genome evolution.

**Figure:**
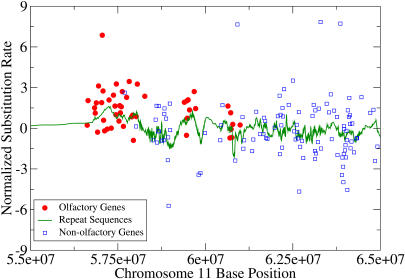
Olfactory genes lie in a mutational “hot spot”

